# Childhood Food Experiences and Selected Eating Styles as Determinants of Diet Quality in Adulthood—A Cross-Sectional Study

**DOI:** 10.3390/nu15102256

**Published:** 2023-05-10

**Authors:** Aleksandra Małachowska, Jerzy Gębski, Marzena Jeżewska-Zychowicz

**Affiliations:** Department of Food Market and Consumer Research, Institute of Human Nutrition Sciences, Warsaw University of Life Sciences (SGGW-WULS), Nowoursynowska 159C, 02-776 Warsaw, Poland

**Keywords:** parental feeding practices, childhood food experiences, eating style, external eating, restrained eating, intuitive eating, diet quality, eating behaviors

## Abstract

Available studies suggest that childhood food experiences (CFE) may be linked with eating behaviors in adulthood, as well as eating style (ES); thus, both CFE and ES can determine dietary intake. Little is known about the role of both of these factors in explaining the diet quality (DQ) of adults. The aim was to investigate to what extent selected ESs, i.e., intuitive (IE), restrained (ResEat), and external (ExtEat) eating, and CFE related to parental feeding practices (PFPs) will predict the DQ of women and men. Data from 708 Polish adults (477 women and 231 men) aged 18–65 were collected via the Internet from October 2022 to January 2023. Mann–Whitney’s U Test was used to compare ES and CFE levels among women and men, while DQ determinants were tested with the multiple linear regression (MLR). In the total study sample, “Healthy Eating Guidance” (CFE), “Child Control” (CFE), “Body–Food Choice Congruence” (IE), and ResEat favored higher DQ scores, while “Unconditional Permission to Eat” (IE), “Eating for Physical Rather Than Emotional Reasons” (IE), and ExtEat predisposed to lower DQ scores. After the MLR was conducted separately in women and men, differences were noted in the role of “Healthy Eating Guidance” (CFE), “Pressure and Food Reward” (CFE), “Unconditional Permission to Eat” (IE), “Eating for Physical Rather Than Emotional Reasons” (IE), ExtEat, and ResEat in predicting DQ indices. Our findings suggest that childhood food experiences and selected eating styles may differently determine the DQ of women and men. Future studies conducted within representative samples are needed to confirm these results.

## 1. Introduction

With the growing prevalence of overweight and obesity observed worldwide [1], long-term effective weight management methods directed at behavioral and lifestyle modifications are in demand [2]. Development of such strategies, for both treatment and prevention purposes, requires an understanding of mechanisms related to one’s increased risk of improper eating behaviors and, thus, a greater probability of excessive body mass [2]. Among several factors, psychological ones have been found to greatly influence eating behaviors [3]. Eating styles (ESs), e.g., intuitive, restrained, or external eating, are described as constructs related to dietary behaviors and involve certain psychological traits, such as food motives, general feelings, and thoughts about food, as well as those which occur in the process of eating [4,5]. Intuitive eating (IE) is believed to represent adaptive eating styles (AESs) and promote eating in response to internal hunger and satiety cues, while restrained (ResEat) and external (ExtEat) eating are examples of maladaptive eating styles (MAESs) that may favor food intake in response to cues unrelated to physical signals [6,7]. 

Available studies suggest that AESs and MAESs may explain the dietary intake of macro- and micronutrients, as well as the intake of favorable (e.g., fruit and vegetables) and unfavorable (e.g., sweets, salty snacks, and fast foods) foods [8,9,10,11,12,13,14,15,16,17]; therefore, they may be useful in determining one’s risk of overweight and obesity [8,9,18,19,20]. Despite indicating relationships between eating styles and food intake, existing studies focused on a single eating style or styles with a similar specificity (e.g., intuitive and mindful) and their association with food intake. Only a few studies included both AESs and MAESs while testing their relationship with food intake [6,21,22]. Among several dietary tools, diet quality indices allow for the assessment of one’s general diet and its alignment with dietary recommendations on the basis of, for example, food intake frequency [23]. The relationship between general diet quality and the mentioned ESs was not thoroughly investigated in previous studies, leaving a research gap [14,23,24,25,26].

The ability to eat in response to internal cues in amounts adequate for the body’s demands, which is characteristic for AESs, is inborn, yet may be disrupted by external factors [27]. Parental influence is a well-known factor influencing children’s eating behavior starting from an early age, i.e., 0–36 months [28]. Parental feeding practices (PFPs) are defined as food- or eating-related strategies used by the parents to impact their children’s eating manner [29]. PFPs, such as encouraging children to try new foods, modeling healthy eating, eating family meals, monitoring children’s hunger and satiety signals, or engaging children in planning and preparing meals [28], may favor healthier eating behaviors, including greater intake of fruit and vegetables, lower intake of energy-dense food, and greater diet quality [30,31,32,33]. As food preferences and eating habits shaped during childhood may persist into adolescence and later into adulthood, early and middle childhood periods, which are greatly influenced by PFPs, are critical for the future way of eating [28]. Several studies have shown that childhood food experiences (CFEs) related to negative PFPs, such as pressure to eat, using food as a reward or punishment, and using qualitative and quantitative restrictions to control children’s body weight, may correlate with greater levels of maladaptive eating in adulthood including excessive food preoccupation, selective eating, long-lasting food rejection, or other disordered eating symptoms, i.e., MAESs [34,35,36,37,38,39,40,41,42]. However, still little is known about the extent to which childhood food experiences may explain food intake or diet quality in adulthood when being simultaneously included with both AESs and MAESs [16,17,22,43].

This study aimed to determine which psychosocial factors allow explaining the diet quality in adulthood to a greater extent among women and men: adults’ memories of childhood food experiences related to parental feeding practices or selected adaptive (i.e., intuitive eating) and maladaptive (i.e., restrained and external eating) eating styles. The following hypotheses were tested:

(1) Childhood food experiences, such as healthy eating guidance, monitoring, and child control, favor greater diet quality in adulthood, while childhood food experiences such as restrictions, food rewarding, or pressuring predispose to less favorable diet quality in adulthood;

(2) Some adaptive and maladaptive eating styles of adults allow explaining the diet quality to a greater extent than childhood food experiences.

## 2. Materials and Methods

### 2.1. Study Design and Sample Collection

A cross-sectional study was conducted in Poland between October 2022 and January 2023 with the use of the CAWI (computer-assisted web interview) technique. The link to the questionnaire was published within diverse Facebook groups. The snowball sampling method was also used to collect more participants. The final study sample consisted of 708 participants (477 women and 231 men) aged 18–65. Each respondent gave voluntary consent to participate in the study. Anonymity of the data, as well as confidentiality, was assured. The Ethics Committee of the Institute of Human Nutrition Sciences, Warsaw University of Life Sciences, in Poland, approved the study design (Resolution No. 02/2020). 

### 2.2. Instruments: Intuitive Eating Scale-2 (IES-2), Dutch Eating Behavior Questionnaire (DEBQ), Adult’s Memories of Feeding in Childhood (AMoFiC)

The Polish version of the Intuitive Eating Scale 2 (IES-2) [16] was used to assess intuitive eating. The Polish version consists of 16 items within four factors: “Reliance on Hunger and Satiety Cues” (six items), “Eating for Physical Rather Than Emotional Reasons” (four items), “Body–Food Choice Congruence” (three items), and “Unconditional Permission to Eat” (three items). Participants were asked to refer to the statements using a five-point Likert scale, ranging from strongly disagree (1) to strongly agree (5). The average score was calculated for each subscale by adding scores obtained from individual items and dividing them by the number of items included in a subscale. 

Restrained and external eating were assessed with the adapted and validated Polish version of the Dutch Eating Behavior Questionnaire (DEBQ) [17]. The “Restrained Eating” subscale consisted of nine items, while “External Eating” was a seven-item subscale. Participants were asked to refer to the statements/questions using a five-point Likert scale, ranging from never (1) to very often (5). The average score was calculated for each subscale by adding scores obtained from individual items and dividing them by the number of items included in a subscale. 

The Adults’ Memories of Feeding in Childhood (AMoFiC) [43] questionnaire was used to assess adults’ memories of childhood food experiences related to parental feeding practices. The AMoFiC consists of the following subscales: “Restrictions” (thirteen items), “Healthy Eating Guidance” (nine items), “Pressure and Food Reward” (six items), “Monitoring” (five items), and “Child Control” (six items). Participants were asked to refer to the statements/questions using a five-point Likert scale, ranging from never (1) to always (5) or from disagree (1) to agree (5), depending on the statement/question type. Participants were also able to choose the answer “I do not remember”, which attracted a score of 0. The average score was calculated for each subscale by adding scores obtained from individual items and dividing them by the number of items included in a subscale. 

### 2.3. Assessment of the Diet Quality

Questions from the Dietary Habits and Nutrition Beliefs Questionnaire (KomPAN) [44] regarding the frequency of the intake of 24 food groups (1. wholemeal (brown) bread/bread rolls; 2. buckwheat, oats, wholegrain pasta, or other coarse-ground groats; 3. milk (including flavored milk, hot chocolate, and latte); 4. fermented milk drinks, e.g., yoghurts and kefir (natural or flavored); 5. fresh cheese curd products, e.g., cottage cheese, cream cheese, and cheese-based puddings; 6. white meat, e.g., chicken, turkey, and rabbit; 7. fish; 8. legume-based foods, e.g., beans, peas, soybeans, and lentils; 9. fruit; 10. vegetables; 11. white bread and bakery products, e.g., wheat bread, rye bread, wheat–rye bread, toast bread, and bread rolls; 12. white rice, white pasta, and fine-ground groats, e.g., semolina and couscous; 13. fast foods, e.g., potato chips/French fries, hamburgers, pizza, and hot dogs; 14. fried foods (e.g., meat or flour-based foods such as dumplings and pancakes); 15. butter as a bread spread or as an addition to your meals for frying/baking etc.; 16. lard as a bread spread or as an addition to your meals for frying/baking, etc.; 17. cheese (including processed cheese and blue cheese); 18. cured meat, smoked sausages, and hot dogs; 19. red meat, e.g., pork, beef, veal, lamb, and game; 20. sweets, e.g., confectionary, biscuits, cakes, chocolate bars, and cereal bars; 21. tinned (jar) meats; 22. sweetened carbonated or still drinks such as Coca-Cola, Pepsi, Sprite, Fanta, and lemonade; 23. energy drinks such as Red Bull, Monster, and Rockstar; 24. alcoholic beverages over the last year. Respondents were asked to relate to the questions on their intake using a six-point Likert scale ranging from never (1) to few times a day (6). Then, the answers were changed into daily frequency (times/day) to enable calculation of the three diet quality indices—nHDI-14, Non-Healthy Diet Index (food groups 11–24); pHDI-10, Pro-Healthy Diet Index (food groups 1–10); DQI, Diet Quality Index (food groups 1–24)—as proposed by Wądołowska and Krusińska [45].

### 2.4. Sociodemographic Characteristics 

The following sociodemographic characteristics were included in the study questionnaire: gender, age (in years), education level (primary, lower secondary, upper secondary, or higher), and place of residence (village, town below 20,000 inhabitants, town between 20,000 and 100,000 inhabitants, or city with over 100,000 inhabitants).

### 2.5. Statistical Analysis

Sociodemographic characteristics of the study sample, i.e., gender, age, education, and place of residence, were presented with the use of descriptive statistics. 

The Shapiro–Wilk test was chosen to check the normality of the distribution. Differences between the factor scores for AMoFiC and IES-2, as well as for ResEat, ExtEat, and three diet quality indices (nHDI-14, pHDI-10, and DQI), in women and men were tested using the Mann–Whitney U test. A *p*-value lower than 0.05 was considered significant.

Three qualitative variables, i.e., diet quality indices pHDI-10, nHDI-14, and DQI, were used as dependent variables (regressors) in the multiple linear regression models. Subscales related to eating styles (RHSC, EPR, B-FCC, UPE, ExtEat, and ResEat), as well as those describing childhood food experiences (Restrictions, Healthy Eating Guidance, Pressure and Food Reward, Monitoring, and Child Control), were selected as explanatory variables (regressands). The coefficients for models were estimated with a division into gender. For each dependent variable, three models (general—total sample, female, and male) were generated, and obtained coefficients were compared. Lastly, only statistically significant (α = 0.05) explanatory variables of models were considered as those with a significant impact on selected diet quality indices (dependent variables).

The analyses were performed using SAS 9.4. statistical package (SAS Institute Inc., Cary, NC, USA) and IBM SPSS Statistics for Windows, version 28.0 (IBM Corp, Armonk, NY, USA).

## 3. Results

### 3.1. Psychosocial Characteristics of the Study Sample

The study included a total of 708 participants with a female majority (67.4%) (Table 1). The mean age of the participants was 36.9 ± 11.5 years.

Table 2 presents the mean scores for childhood food experiences (CFE) and intuitive eating factors, as well as for restrained and external eating. CFEs were most related to “Healthy Eating Guidance” and least related to “Restrictions”; however, after taking gender into account, no significant differences were observed. Women scored significantly higher in ResEat and ExtEat scales, while a greater score for EPR was noted among men.

Diet quality indices calculated on the basis of the frequency of selected food groups’ intake are presented in Table 3. Men had higher scores for nHDI-14 but scored lower in pHDI-10 and DQI in comparison to women (*p* < 0.001).

### 3.2. Determinants of the Diet Quality 

Predictors of the diet quality indices including childhood feeding experiences and selected eating styles were tested with multiple linear regression (Table 4, Table 5 and Table 6). 

In the total sample, B-FCC, UPE, ExtEat, and ResEat determined nHDI-14 (Table 4). Among women, nHDI-14 was found to be positively correlated with UPE (B = 1.238, *p* = 0.021), ExtEat (B = 0.99, *p* = 0.044), and “Healthy Eating Guidance” (B = 0.879, *p* = 0.046) but negatively correlated with B-FCC (B = −2.886, *p* ≤ 0.001) (Table 4). Among men, nHDI-14 correlated positively with UPE (B = 2.75, *p* = 0.002) and “Pressure and Food Reward” (B = 1.587, *p* = 0.020), but negatively with B-FCC (B = −1.91, *p* = 0.031).

In the total sample, “Healthy Eating Guidance”, “Child Control”, EPR, B-FCC, UPE, and ResEat predicted pHDI-10 score (Table 5). B-FCC was found to be a determinant of pHDI-10 separately in women and men (B = 2.910, *p* ≤ 0.001 and B = 2.005, *p* = 0.043, respectively) (Table 5). Moreover, among women, pHDI-10 correlated negatively with EPR (B = −0.972, *p* = 0.050) and UPE (B = −1.905, *p* = 0.015). ResEat was found to be a predictor of pHDI-10 solely in men (B = 2.418, *p* = 0.012). 

EPR, B-FCC, UPE, and ResEat predicted DQI score in the total sample (Table 6). Analogously to pHDI-10, B-FCC correlated positively with DQI separately in women and men (B = 5.796, *p* ≤ 0.001 and B = 3.915, *p* = 0.003, respectively) (Table 6). Negative correlations between DQI and UPE were observed for women and men (B = −3.143, *p* = 0.001 and B = −3.512, *p* = 0.006, respectively). ResEat determined the DQI of men, as a positive correlation between these variables was noted (B = 2.905, *p* = 0.013).

## 4. Discussion

We aimed to examine to what extent childhood food experiences (CFEs) and selected eating styles (ESs), i.e., intuitive (IE), restrained (ResEat), and external (ExtEat) eating, are useful in explaining the diet quality of Polish adults. The previously conducted research did not provide sufficient evidence on the role of the CFE on future eating behaviors with a particular focus on gender differences [34,35,36,37,38,39,40,41,42].

Our first hypothesis that selected CFEs related to the parental feeding practices (PFPs) favor worse or greater diet quality in adulthood was only partially confirmed. It turned out that CFEs did not differentiate the general Diet Quality Index (DQI), while differences were noted in pHDI-10 score after considering the “Healthy Eating Guidance” and “Child Control” subscales. Such experiences predicted greater scores for pHDI-10 in the total sample. “Healthy Eating Guidance” relates to, for example, modeling healthy eating, involving child in food preparation process, or encouraging balance and variety, as well as keeping mostly healthy food in house [43]. The latter can be viewed as an example of covert control [28]. During middle childhood, this strategy can be beneficial as the child is less likely to detect such control [28]. However, it may be questioned how it affects future eating behaviors, starting from adolescence, when parents have less control over the child’s diet and a child has greater access to diverse foods, including those previously absent at home [43]. Parent-controlled availability of products commonly identified as unhealthy may be more beneficial in the long run than a strategy of avoiding them on a daily basis, which can lead to a “forbidden fruit effect” [46]. Nevertheless, no significant association was found between “Restrictions” and all three diet quality indices. Among women solely, “Healthy Eating Guidance” was positively correlated with nHDI-14. Girls tend to be more often engaged in the meal preparation process than boys, which may favor greater diet quality in childhood and adolescence [28,30,31,32,33,47]. However, a longitudinal study found that involving adolescents in cooking does not predict a better diet quality in young adulthood [48]. A possible explanation refers to the increased prevalence in dieting and disordered eating observed in the female adolescents [49]. Such unhealthy dietary practices may negatively affect the self-regulation of food intake [19,50], thus causing poorer diet quality [49], which is consistent with our findings. Furthermore, there is a possibility that maternal ESs might affect daughters’ ESs differently than sons’ ESs, especially in terms of ResEat, leading to an increased interest in dieting [5]. 

“Child Control” was positively correlated with DQI in the total sample. In contrary to control food rules applied by the parents [34], “Child Control” measured by the Adults’ Memories of Feeding in Childhood questionnaire (AMoFiC) relates to a different type of control, i.e., being responsive to the child’s needs and demands [43]. Nevertheless, some level of parental control over child’s eating, e.g., food choices and availability of snacks throughout the day, may be beneficial as permissive/indulgent or neglecting parenting is related to less favorable eating behaviors in children and a higher risk of overweight or obesity [51]. Little is known about the role of moderate parental control as an alternative to strict parental control or lack of control; thus, this aspect should be included in future studies [52]. The role of parental control as a trigger of food experiences remains unclear and requires further research [43,53,54]. 

Childhood experiences of “Pressure and Food Reward” predisposed only men to the greater score in nHDI-14. These findings may be explained by the gender differences in food-reward processing. It is suggested that, among men, eating provides a greater hedonic effect, and the satiation after finishing the meal delivers a more a rewarding feeling [55]. Harris et al.’s study among 3–4 year old children suggested that the pressure to eat might be positively linked to eating in the absence of hunger, yet only among boys [56]. Another study found that, in adolescent boys only, a higher exposure to pressuring can predispose to the greater intensity of unhealthy and extreme weight control behaviors [57]. Moreover, for middle-aged boys, the perceived pressure to eat was suspected to impair adequate self-regulation of food intake as it favored both external and emotional eating [58]. The abovementioned research suggests that pressuring can have different impact on girls and boys and, possibly, eating behaviors in adulthood, which is coherent with our findings. Episodes of forced consumption of novel, disliked, or aversive foods in childhood may lower the intake of the target food in adulthood [39]. Despite being effective short-term, using food as a reward or punishment can trigger greater preferences for unhealthy foods, leading to the increased risk of not meeting dietary recommendations for healthy foods intake, including vegetables or protein-rich foods [34], which supports our results. 

Our study did not take into account the possible diverse impact of both parents, mothers, and fathers, and hypothetical different influence on girls’ and boy’s eating behaviors [5,59]. It is also possible that other caregivers, e.g., other family members, had an impact on the child’s nutrition and, thus, possible future eating behaviors [60]. These limitations might have affected our results regarding men and women. Moreover, available longitudinal studies tracking changes in dietary patterns (DP) from childhood to adolescence or young adulthood proved that DP established in childhood only moderately continue in adulthood, which supports our findings that only selected CFEs determined diet quality [61,62,63]. It may be also assumed that CFEs in a greater manner influence long-term eating style rather than eating habits or diet quality [34,35,36,37,38,39,40,41,42]. More longitudinal studies would allow for a greater understanding of this process; however, tracking changes in ES and dietary intake for such a long period of time may be difficult [61,62,63]. Another possible factor that could have influenced our findings is that the study participants scored generally low in all AMoFiC subscales. Future research in this area among participants who experienced PFPs in childhood to a greater extent, along with the use of different measures, is suggested.

The hypothesis that selected ESs will determine diet quality to a greater extent than CFEs was confirmed. It appeared that “Body–Food Choice Congruence” (B-FCC), which is an IE component, predicted diet quality to the greatest extent by favoring higher scores for pHDI-10 and DQI and a lower score for nHDI-14 for the total study sample, as well as among men and women separately, which is in line with the available studies [23,24]. Similarly to the previous findings [14,23,26], “Unconditional Permission to Eat” (UPE) was related to lower diet quality in our study. In the total sample and separately among women and men, positive correlations with pHDI-10 and DQI and negative correlations with nHDI-14 were noted, with one exception—a lack of correlation with pHDI-10 in men. Eating in response to internal cues and choosing the food that is desired [64] might be problematic for people who restricted intake of “unhealthy foods” or their general caloric intake in the past [16]. Dieting may disrupt one’s ability to adequately self-regulate food intake during the lifespan [27,64]. A prolonged period of restriction can favor a higher level of disinhibited eating, including external eating; it may provoke the feeling of deprivation and food preoccupation, possibly resulting in a greater intake of previously prohibited foods [8,16,19,64]. An adequate understanding of body signals and a rejection of the diet mentality may be challenging, especially in the initial phase of IE and among individuals who engaged in restrictions in the past [64]. Due to our observations, UPE may cause greater intake of unfavorable foods, yet this effect may be only temporarily observed in the food intake [16]. Hypothetically, it may constitute one of the reasons why the results of selected IE interventions studies aimed at diet quality improvement are inconsistent [65]. UPE was not found to be a predictor of pHDI-10 among men only. A possible explanation relates to the fact that men tend to restrict food intake to a lower extent than women [17], which was also supported by the difference in the ResEat score of men and women in our study. Therefore, the abovementioned mechanism may be characteristic and more common among women [19]. Our findings regarding “Eating for Physical Rather Than Emotional Reasons” (EPR), which correlated negatively with pHDI-10 and DQI in the total study sample and additionally with pHDI-10 solely in women, may also be partially explained by the above-described temporary mechanism. The state of hunger may affect mood-related food cravings, specifically in a form of high-density foods [66]. Intuitive eaters should, therefore, avoid longer periods of fasting and, thus, a ravenous state, which can influence less favorable food choices [64]. The use of the IES-2 allows examining the level of IE components, yet incoherent studies and previously mentioned uncertainties have suggested that examining the relationship between IE and food intake/diet quality requires more comprehensive insight into psychosocial determinants of the diet quality. Future research should include not only IE measures, i.e., IES-2, but also additional questions regarding previous periods of restrictions and the ability to detect hunger/satiety [16].

ExtEat explained the diet quality indices to a lower extent (nHDI-14) than ResEat (nHDI-14, pHDI-10, and DQI), yet ExtEat scores were higher than ResEat ones, suggesting higher food intake due to environmental cues in the study sample [7]. However, measuring ExtEat with the DEBQ [7] does not provide information about the frequency of those environmental stimuli, which might have affected our results [11]. ExtEat is suggested to be a weak predictor of body mass index [17,18]. However, our findings indicated that a higher score in ExtEat predisposes to a higher score in nHDI-14, which may consequently promote weight gain. Despite the fact that ExtEat is believed to be more useful in explaining the occasional food intake rather than the overall dietary intake [8,11], the obtained results show the utility of ExtEat in explaining the overall quality of the diet. ExtEat correlated positively with nHDI-14 in the group of women, while no such relationship was observed in the group of men. External eating is related to food cravings [67,68], and greater food cravings has been shown in females compared with males [69]. Thus, the differences in food craving in men and women reflecting externality [67] may explain the differences in relationship between ExtEat and nHDI-14 in women and men. Moreover, greater craving for sweet foods in females suggest that sweet foods as a part of nHDI-14 might be crucial in appetite control in females [67]. The response to a variety of external cues (ExtEat) is heightened in restrained eaters [8], and females scored higher on restrained eating compared with males [10].

ResEat on the other hand correlated negatively with nHDI-14 and positively with pHDI-10 and DQI in the total sample. Our findings suggest that ResEat may favor healthier eating habits including greater intake of favorable foods [25]. The same findings were observed in the male group only, which supports the need to search for more underlying mechanisms between dieting and diet quality separately among women and men [19,25,70]. Despite the positive consequences of ResEat, i.e., positive correlations with pHDI-10 and DQI and negative correlation with nHDI-14, it should be considered that prolonged periods of restrictions may be counterproductive and result in weight gain as it increases one’s chances of disinhibited eating, e.g., uncontrolled episodes of eating, emotional eating, and binge eating episodes [19,50]. Assuming that ResEat might lead to disturbed eating when used for a longer period of time, further studies that would enable detecting any possible changes in ResEat level and diet quality from baseline to follow-up are suggested [19]. The complexity of the ResEat construct suggests that tools other than the DEBQ to measure ResEat should also be used to comprehensively determine its relationship with the diet quality [70].

The differences in diet quality indices between men and women in our sample are in line with other research. The results of a global study [71] showed that men scored significantly lower in pHDI-10 and DQI and higher in nHDI-14 in comparison to women. These differences can be explained using both childhood food experiences and ESs. The level of “Pressure and Food Reward” did not differ among women and men. Nevertheless, it predicted diet quality of men solely, suggesting that the experience of this CFE may be a key factor in male food choices [34,39]. ResEat in men predicted greater scores for pHDI-10 and DQI. Therefore, a lower level of ResEat in men may have influenced worse diet quality [25]. Another explanation could be the fact that EPR was significantly higher in men in comparison to women in our study. Although our results, similarly to previous ones [14,24,26], do not confirm the link between EPR and diet quality, Lopez et al. study [23] suggested that EPR may positively correlate with the diet quality. It seems that the observed differences in the levels of CFEs and ESs may explain the worse diet quality of men. However, as the previous studies found, other sociodemographic and lifestyle factors (e.g., age and education level) may also be useful for that purpose [17,71]. Another important diet quality determinant, which was not included in the current research, is socioeconomic status (SES). Available research has shown that low SES may predispose to a less healthy diet, while higher SES can favor greater compliance with dietary guidelines [72]. Future research should test whether the relationship among CFE, ES, and diet quality differs depending on the SES.

Our results contribute to the increase in knowledge about the mechanisms affecting diet quality. This study allowed identifying some differences in determinants of diet quality in adult women and men, confirming the need to include intergender differences in further research. The findings regarding intuitive eating can be useful for other researchers planning further studies on its long-term effectivity and its relationships with other eating styles and indicators of physical and psychological health, as well as dietary intake. Moreover, results regarding the influence of childhood food experiences enabled recognizing most significant parental feeding practices that determine future eating behaviors, thus providing guidelines for future research and confirming their importance.

### Strengths and Limitations

To our knowledge, this is the first study to include both selected adaptive and maladaptive eating styles, as well as childhood feeding experiences, as predictors of diet quality. Moreover, our study allowed comparing diet quality determinants of women and men and detecting any possible differences. Questions regarding the frequency of food intake used for the calculation of the diet quality indices were related to the last 12 months of consumption; thus, possible seasonal changes in food intake were included. Nevertheless, several limitations should be mentioned. The cross-sectional character of the study did not allow determining causality. The study sample was not representative of the Polish population; thus, the study results cannot be generalized. Another limitation relates to the assessment of the childhood feeding experiences, which did not take into account the separate role of mothers and fathers or other caregivers. Moreover, the findings concerning childhood food experiences might have been biased by imprecise recollections of participants. Therefore, longitudinal cohort studies tracking the impact of parental feeding practices from birth to adulthood are suggested to confirm our results. This would allow for greater insight into the role of the parental feeding practices on diet quality and enable early intervention in childhood. The fact that only selected adaptive and maladaptive eating styles were measured may be highlighted as another limitation. Lastly, the study did not include other possible determinants of the diet quality, such as socioeconomic and health status.

## 5. Conclusions

Our findings showed that childhood food experiences related to parental feeding practices and selected eating styles, i.e., intuitive, restrained, and external eating, may explain the diet quality of adults, along with eating styles, in a more comprehensive way. Only childhood food experiences related to “Healthy Eating Guidance” and “Child Control” parental practices were found to determine diet quality in adulthood. The tested eating styles were found to predict diet quality of the participants in a different manner, with mixed results related to the intuitive eating domains. “Healthy Eating Guidance”, “Child Control”, “Body-Food Choice Congruence” (intuitive eating), and “Restrained Eating” favored greater diet quality, while “Unconditional Permission to Eat” (intuitive eating), “Eating for Physical Rather Than Emotional Reasons” (intuitive eating), and “External Eating” predisposed to lower scores of the diet quality indices. Intergender differences in factors determining diet quality were noted. Among women, “Healthy Eating Guidance” and “External Eating” were positively correlated with the Non-Healthy Diet Index, while “Unconditional Permission to Eat” and “Eating for Physical Rather Than Emotional Reasons” were negatively linked to the Pro-Healthy Diet Index. In the male group, “Restrained Eating” was found to be positively correlated with the Pro-Healthy Diet Index and Diet Quality Index, whereas “Pressure and Food Reward” predisposed to a greater score of the Non-Healthy Diet Index.

The results contribute to both research and application, allowing for their implementation in creating dietary interventions aimed at the improvement of eating behaviors and weight outcomes in children and adults. Our findings regarding the role of childhood food experiences may be used in planning educational programs for adults focused on the use of diverse parental feeding practices, their influence on the development of children’s eating behaviors, and their possible long-term effect on the future eating behaviors of their children. The findings related to eating styles can be included in education activities and dietary management aimed at the change of unfavorable eating behaviors into positive ones, including the promotion of greater diet quality and the ability for adequate, self-regulated dietary intake. It is suggested that future studies test for the role of childhood food experiences as mediators or moderators of the relationship between eating styles and diet quality within representative samples, taking into account differences between women and men.

## Figures and Tables

**Table 1:** Sociodemographic characteristics of the study sample.

Variables		Total (N = 708) N (%)	Women (N = 477) N (%)	Men (N = 231) N (%)
**Age** **(years)**	18–24	94 (13.3)	65 (13.6)	29 (12.6)
	25–39	349 (49.3)	253 (53.0)	96 (41.6)
	40–54	193 (27.3)	120 (25.2)	73 (31.6)
	55–65	72 (10.2)	39 (8.2)	33 (14.3)
**Education**	Primary	3 (0.4)	3 (0.6)	0 (0.0)
	Lower secondary	3 (0.4)	2 (0.4)	1 (0.4)
	Upper secondary	113 (16.0)	68 (14.3)	45 (19.5)
	Higher (e.g., BSc and MSc)	589 (83.2)	404 (84.7)	185 (80.1)
**Place of Residence**	Village	113 (16.0)	78 (16.4)	35 (15.2)
	Town below 20,000 inhabitants	43 (6.1)	31 (6.5)	12 (5.2)
	Town between 20,000 and 100,000 inhabitants	109 (15.4)	69 (14.5)	40 (17.3)
	City with over 100,000 inhabitants	443 (62.6)	299 (62.7)	144 (62.3)

N, number of participants; BSc, Bachelor of Science; MSc, Master of Science.

**Table 2:** Childhood food experiences and eating styles in the study sample.

Subscales	Total (N = 708) M ± SD	Women (N = 477) M ± SD	Men (N = 231) M ± SD
Restrictions ^1^	1.93 ± 0.60	1.94 ± 0.61	1.93 ± 0.56
Healthy Eating Guidance ^1^	3.21 ± 0.90	3.22 ± 0.93	3.19 ± 0.84
Pressure and Food Reward ^1^	2.62 ± 0.94	2.64 ± 0.97	2.58 ± 0.87
Monitoring ^1^	2.57 ± 1.06	2.60 ± 1.09	2.50 ± 0.98
Child Control ^1^	2.55 ± 0.78	2.60 ± 0.81	2.46 ± 0.70
RHSC ^2^	3.40 ± 0.81	3.36 ± 0.85	3.46 ± 0.71
EPR *** ^2^	3.33 ± 1.15	3.13 ± 1.16	3.75 ± 0.99
B-FCC ^2^	3.43 ± 0.78	3.44 ± 0.81	3.41 ± 0.71
UPE ^2^	3.63 ± 0.82	3.60 ± 0.83	3.67 ± 0.79
ExtEat ** ^3^	2.97 ± 0.72	3.04 ± 0.73	2.85 ± 0.67
ResEat *** ^3^	2.62 ± 0.88	2.70 ± 0.88	2.45 ± 0.86

N, number of participants; M, mean; SD, standard deviation; ^1^ Adults’ Memories of Feeding in Childhood (each subscale range: 0–5); ^2^ Intuitive Eating Scale 2 (each subscale range: 1–5); ^3^ Dutch Eating Behavior Questionnaire (selected subscales; each subscale range: 1–5); RHSC, Reliance on Hunger and Satiety Cues; EPR, Eating for Physical Rather Than Emotional Reasons; B-FCC, Body–Food Choice Congruence; UPE, Unconditional Permission to Eat; ResEat, Restrained Eating; ExtEat, External Eating; significant at ** *p* < 0.01, *** *p* < 0.001; Mann–Whitney U test.

**Table 3:** Diet quality in the study sample.

Diet Quality Indices	Total (N = 708)		Women (N = 477)		Men (N = 231)	
	M ± SD	Min–Max	M ± SD	Min–Max	M ± SD	Min–Max
nHDI-14 ***	14.90 ± 8.44	0.21–54.79	13.82 ± 7.90	0.64–54.79	17.15 ± 9.07	0.21–54.29
pHDI-10 ***	24.26 ± 11.11	1.20–66.40	25.84 ± 10.97	2.00–66.40	20.99 ± 10.69	1.20–56.40
DQI ***	9.35 ± 14.25	−41.49–62.69	12.02 ± 13.74	−20.99–62.69	3.85 ± 13.73	−41.49–44.13

N, number of participants; M, mean; SD, standard deviation; nHDI-14, Non-Healthy Diet Index (range: 0–100 points); pHDI-10, Pro-Healthy Diet Index (range: 0–100 points); DQI, Diet Quality Index (range: −100–100 points); min, minimum value; max, maximum value; significant at *** *p* < 0.001; Mann–Whitney U Test.

**Table 4:** Determinants of the Non-Healthy Diet Index (nHDI-14) in the study sample.

	Total (N = 708)			Women (N = 477)			Men (N = 231)		
	B (SE)	t	*p*	B (SE)	t	*p*	B (SE)	t	*p*
Intercept	10.207 (3.63)	2.81	0.005	12.459 (4.17)	2.99	0.003	2.018 (6.96)	0.29	0.772
Restrictions ^1^	0.916 (0.62)	1.48	0.140	0.626 (0.69)	0.91	0.365	0.38 (1.28)	0.30	0.767
Healthy Eating Guidance ^1^	0.596 (0.40)	1.48	0.139	0.879 (0.44)	2.00	0.046	−0.007 (0.85)	−0.01	0.994
Pressure and Food Reward ^1^	0.368 (0.32)	1.14	0.253	−0.079 (0.35)	−0.22	0.824	1.587 (0.68)	2.34	0.020
Monitoring ^1^	−0.315 (0.36)	−0.89	0.376	−0.318 (0.39)	−0.82	0.414	0.003 (0.75)	0.00	0.997
Child Control ^1^	0.426 (0.38)	1.12	0.265	0.624 (0.41)	1.50	0.134	0.309 (0.83)	0.37	0.709
RHSC ^2^	0.353 (0.44)	0.8	0.422	0.644 (0.5)	1.30	0.196	0.285 (0.87)	0.33	0.743
EPR ^2^	0.323 (0.31)	1.04	0.297	−0.349 (0.35)	−0.99	0.323	0.871 (0.65)	1.34	0.181
B-FCC ^2^	−2.742 (0.44)	−6.24	<0.001	−2.886 (0.49)	−5.86	<0.001	−1.91 (0.88)	−2.17	0.031
UPE ^2^	1.678 (0.46)	3.66	<0.001	1.238 (0.53)	2.32	0.021	2.75 (0.85)	3.22	0.002
ExtEat ^3^	1.02 (0.48)	2.13	0.033	0.99 (0.54)	1.86	0.044	1.022 (0.96)	1.07	0.286
ResEat ^3^	−0.845 (0.43)	−1.96	0.050	−0.702 (0.51)	−1.38	0.167	−0.486 (0.79)	−0.62	0.537

N, number of participants; B, parameter estimate; SE, standard error; t, test statistic; *p*, probability value; ^1^ Adults’ Memories of Feeding in Childhood; ^2^ Intuitive Eating Scale 2; ^3^ Dutch Eating Behavior Questionnaire (selected subscales); RHSC, Reliance on Hunger and Satiety Cues; EPR, Eating for Physical Rather Than Emotional Reasons; B-FCC, Body–Food Choice Congruence; UPE, Unconditional Permission to Eat; ResEat, Restrained Eating; ExtEat, External Eating.

**Table 5:** Determinants of the Pro-Healthy Diet Index (pHDI-10) in the study sample.

Variables	Total (N = 708)			Women (N = 477)			Men (N = 231)		
	B (SE)	t	*p*	B (SE)	t	*p*	B (SE)	t	*p*
Intercept	10.803 (4.94)	2.19	0.029	13.615 (6.10)	2.23	0.026	4.417 (8.49)	0.52	0.603
Restrictions ^1^	0.360 (0.84)	0.43	0.670	1.02 (1.01)	1.01	0.313	−0.173 (1.56)	−0.11	0.912
Healthy Eating Guidance ^1^	1.126 (0.55)	2.06	0.040	1.005 (0.64)	1.57	0.118	0.865 (1.04)	0.83	0.407
Pressure and Food Reward ^1^	−0.092 (0.44)	−0.21	0.834	−0.316 (0.52)	−0.61	0.541	0.896 (0.83)	1.08	0.280
Monitoring ^1^	−0.6 (0.48)	−1.24	0.215	−0.527 (0.57)	−0.93	0.355	−1.227 (0.91)	−1.35	0.180
Child Control ^1^	0.859 (0.52)	1.65	0.049	0.757 (0.61)	1.25	0.213	0.651 (1.01)	0.65	0.520
RHSC ^2^	0.305 (0.60)	0.51	0.609	0.666 (0.73)	0.92	0.360	−0.263 (1.06)	−0.25	0.804
EPR ^2^	−0.963 (0.42)	−2.29	0.023	−0.972 (0.52)	−1.89	0.050	0.496 (0.79)	0.63	0.532
B-FCC ^2^	2.727 (0.60)	4.56	<0.001	2.91 (0.72)	4.04	<0.001	2.005 (1.07)	1.87	0.043
UPE ^2^	−1.446 (0.62)	−2.32	0.021	−1.905 (0.78)	−2.44	0.015	−0.762 (1.04)	−0.73	0.466
ExtEat ^3^	1.174 (0.65)	1.80	0.072	1.301 (0.79)	1.65	0.099	0.846 (1.17)	0.73	0.469
ResEat ^3^	1.259 (0.58)	2.15	0.032	0.35 (0.74)	0.47	0.637	2.418 (0.96)	2.52	0.012

N, number of participants; B, parameter estimate; SE, standard error; t, test statistic; *p*, probability value; ^1^ Adults’ Memories of Feeding in Childhood; ^2^ Intuitive Eating Scale 2; ^3^ Dutch Eating Behavior Questionnaire (selected subscales); RHSC, Reliance on Hunger and Satiety Cues; EPR, Eating for Physical Rather Than Emotional Reasons; B-FCC, Body–Food Choice Congruence; UPE, Unconditional Permission to Eat; ResEat, Restrained Eating; ExtEat, External Eating.

**Table 6:** Determinants of the Diet Quality Index (DQI) in the study sample.

Variables	Total (N = 708)			Women (N = 477)			Men (N = 231)		
	B (SE)	t	*p*	B (SE)	t	*p*	B (SE)	t	*p*
Intercept	0.596 (5.97)	0.1	0.921	1.156 (7.20)	0.16	0.873	2.399 (10.23)	0.23	0.815
Restrictions ^1^	−0.556 (1.02)	−0.55	0.586	0.394 (1.19)	0.33	0.741	−0.553 (1.88)	−0.29	0.770
Healthy Eating Guidance ^1^	0.53 (0.66)	0.80	0.423	0.126 (0.76)	0.17	0.868	0.872 (1.26)	0.69	0.488
Pressure and Food Reward ^1^	−0.46 (0.53)	−0.87	0.385	−0.237 (0.61)	−0.39	0.697	−0.691 (1.00)	−0.69	0.490
Monitoring ^1^	−0.285 (0.58)	−0.49	0.626	−0.208 (0.67)	−0.31	0.756	−1.23 (1.10)	−1.12	0.265
Child Control ^1^	0.433 (0.63)	0.69	0.490	0.134 (0.72)	0.19	0.852	0.342 (1.22)	0.28	0.779
RHSC ^2^	−0.047 (0.72)	−0.07	0.948	0.022 (0.86)	0.03	0.980	−0.548 (1.28)	−0.43	0.669
EPR ^2^	−1.286 (0.51)	−2.53	0.012	−0.623 (0.61)	−1.02	0.306	−0.375 (0.96)	−0.39	0.695
B-FCC ^2^	5.469 (0.72)	7.57	<0.001	5.796 (0.85)	6.81	<0.001	3.915 (1.29)	3.03	0.003
UPE ^2^	−3.124 (0.75)	−4.15	<0.001	−3.143 (0.92)	−3.41	0.002	−3.512 (1.26)	−2.79	0.006
ExtEat ^3^	0.154 (0.79)	0.20	0.845	0.301 (0.93)	0.32	0.746	−0.176 (1.41)	−0.13	0.901
ResEat ^3^	2.104 (0.71)	2.98	0.003	1.052 (0.88)	1.20	0.230	2.905 (1.16)	2.51	0.013

N, number of participants; B, parameter estimate; SE, standard error; t, test statistic; *p*, probability value; ^1^ Adults’ Memories of Feeding in Childhood; ^2^ Intuitive Eating Scale 2; ^3^ Dutch Eating Behavior Questionnaire (selected subscales); RHSC, Reliance on Hunger and Satiety Cues; EPR, Eating for Physical Rather Than Emotional Reasons; B-FCC, Body–Food Choice Congruence; UPE, Unconditional Permission to Eat; ResEat, Restrained Eating; ExtEat, External Eating.

## Data Availability

The data presented in this study are available on request from the corresponding author.
